# Sterility maintenance assessment of moist/wet material after steam sterilization and 30-day storage

**DOI:** 10.1186/1753-6561-5-S6-P316

**Published:** 2011-06-29

**Authors:** GADA Moriya, KU Graziano

**Affiliations:** 1 Departamento de Enfermagem Médico-Cirúrgica, Nursing School – University of Sao Paulo, São Paulo, Brazil; 2 CSSD, Hospital Alemão Oswaldo Cruz, São Paulo, Brazil

## Introduction / objectives

Moist/wet materials stored after autoclaving are considered contaminated and not recommended for use. Aiming to support decision-making in emergency situations, this study evaluated the maintenance of sterility in moist/wet material after being submitted to steam sterilization and stored for a period of 30 days.

## Methods

As carriers to be incubated in culture medium for proof of sterility, 1600 porcelain cylinders were attached in surgical instruments. The surgical instruments were inserted into boxes following the recommended surgical care practices. 40 surgical boxes packed in nonwoven cloth covering Spunbound, Metblouwn, Spunbound (SMS): half (the experimental group) were placed in an autoclave but the drying phase was interrupted, yielding moist/wet materials and the other half (the negative control group) underwent the complete cycle. The external parts of each surgical box were deliberately contaminated with Serratia marcescens and subsequently stored for 30 days.

## Results

The presence of moisture within the surgical boxes were confirmed by differential weight of the boxes before and after autoclaving. After storage, the boxes' contents were submitted to sterility tests and no microbiologial growth was observed.

## Conclusion

The presence of moisture inside the surgical boxes did not interfere with maintaining their sterility after deliberated external contamination and 30-day storage. This study did not intend to contradict the current recommendations, but only bring scientific evidence to assist in decision making for emergency situations.

## Disclosure of interest

None declared.

